# Association between glucocorticoids treatment and viral clearance delay in patients with COVID-19: a systematic review and meta-analysis

**DOI:** 10.1186/s12879-021-06548-z

**Published:** 2021-10-14

**Authors:** Jianbo Li, Xuelian Liao, Yue Zhou, Luping Wang, Hang Yang, Wei Zhang, Zhongwei Zhang, Yan Kang

**Affiliations:** 1 Department of Critical Care Medicine, West China Hospital, Sichuan University, 37 Guo Xue Xiang St, Chengdu, 610041 Sichuan China; 2 Molecular Medicine Research Center, State Key Laboratory of Biotherapy/Collaborative Innovation Center for Biotherapy, West China Hospital, Sichuan University, 37 Guo Xue Xiang St, Chengdu, 610041 Sichuan China

**Keywords:** COVID-19, Glucocorticoids treatment, Viral clearance delay

## Abstract

**Background:**

Evidence of glucocorticoids on viral clearance delay of COVID-19 patients is not clear.

**Methods:**

In this systematic review and meta-analysis, we searched for studies on Medline, Embase, EBSCO, ScienceDirect, Web of Science, Cochrane Library, and ClinicalTrials.gov from 2019 to April 20, 2021. We mainly pooled the risk ratios (RRs) and mean difference (MD) for viral clearance delay and did subgroup analyses by the severity of illness and doses of glucocorticoids.

**Results:**

38 studies with a total of 9572 patients were identified. Glucocorticoids treatment was associated with delayed viral clearance in COVID-19 patients (adjusted RR 1.52, 95% CI 1.29 to 1.80, I^2^ = 52%), based on moderate-quality evidence. In subgroup analyses, risk of viral clearance delay was significant both for COVID-19 patients being mild or moderate ill (adjusted RR 1.86, 95% CI 1.35 to 2.57, I^2^ = 48%), and for patients of being severe or critical ill (adjusted RR 1.59, 95% CI 1.23 to 2.07, I^2^ = 0%); however, this risk significantly increased for patients taking high doses (unadjusted RR 1.85, 95% CI 1.08 to 3.18; MD 7.19, 95% CI 2.78 to 11.61) or medium doses (adjusted RR 1.86, 95% CI 0.96 to 3.62, I^2^ = 45%; MD 3.98, 95% CI 3.07 to 4.88, I^2^ = 4%), rather those taking low doses (adjusted RR 1.38, 95% CI 0.94 to 2.02, I^2^ = 59%; MD 1.46, 95% CI −0.79 to 3.70, I^2^ = 82%).

**Conclusions:**

Glucocorticoids treatment delayed viral clearance in COVID-19 patients of taking high doses or medium doses, rather in those of taking low doses of glucocorticoids.

**Supplementary Information:**

The online version contains supplementary material available at 10.1186/s12879-021-06548-z.

## Introduction

As of early August 2021, nearly 200 million people have been confirmed with COVID-19, and more than 4 million died according to WHO official updates of global data on COVID-19 [1]. Historically, glucocorticoids were widely recommended to treat SARS, but this proved to be controversial. A recent large randomized controlled trial (RCT) [2] from the United Kingdom compared 2104 hospital COVID-19 patients who were given dexamethasone with those of 4321 patients who were not. Results from this large trial showed glucocorticoid treatment cut the risk of death from 40 to 28% for patients on ventilators and from 25 to 20% for patients needing oxygen. Then, a systematic review and meta-analysis [3] involving 7 RCTs also revealed a significant association between glucocorticoids treatment and decreased mortality in COVID-19 patients of critical illness. Although these results are encouraging, glucocorticoids theoretically delay virus removal. At present, no study has systematically assessed glucocorticoids treatment effects on viral clearance for COVID-19. Thus, we conducted this systematic review and meta-analysis to evaluate this potential effect from glucocorticoids treatment for COVID-19.

## Methods

### Guidance and protocol

We reported our study according to standards of the meta-analysis of observational studies in epidemiology (MOOSE) [4] and preferred reporting items for systematic reviews and meta-analyses (PRISMA) [5]. We registered our protocol for this review and meta-analysis on PROSPERO (CRD42020194225).

### Eligibility criteria and definitions

We considered criteria of eligible studies as follows: participants were COVID-19 patients infected with SARS-CoV-2 confirmed through the nucleic acid test; the intervention was glucocorticoids, no matter types, and doses; the controls were COVID-19 patients receiving usual care except glucocorticoids treatment; the outcomes should involve viral clearance, no matter what kind of data was presented. Both RCTs and observational studies (including cohort studies, case–control studies, case series of more than 10 patients) were included. Viral clearance delay was defined as the opposite of SARS-CoV-2 RNA shedding at any time from illness onset (different studies were based on different time frames, usually at ≥ 7-day from illness onset) and the SARS-CoV-2 RNA shedding was defined as two consecutive RNA negative with at least 24-h intervals and the date of the first negative test was defined as the day of viral RNA clearance [6–9].

### Literature search

Two of the authors (JB.L. and XL.L.) conducted a literature search on several databases: Medline (Ovid), Embase (Ovid), EBSCO, ScienceDirect, Web of Science (All database), Cochrane Library, and ClinicalTrials.gov from 2019 to April 20, 2021. Also, we reviewed reference lists of identified studies, systematic reviews, and review articles on the same topic. Language or publication status was not restricted. Additional file 1: Table S1 showed the details of the search strategy.

### Study selection

After duplicates were removed, the title and abstract of each item were browsed to screen studies with eligible participants and intervention by two independent groups of four authors (H.Y. and W.Z.; Y.Z. and LP.W.). Further screening was conducted to determine whether the item met the rest eligibility criteria. The time, hospital, and number of patients involved in each study were also examined, and studies with highly repetitive cohort and no additional subgroup information would be excluded. Disagreements were resolved by consensus, and if necessary, consultation with a third author (ZW.Z.).

### Data collection process

Data from included studies were extracted into standard collection forms and information tables for quality assessment were created. The quantile estimation method was applied to estimate the sample mean and standard deviation if a study presented summary statistics as median, first and third quartiles, and sample size. Note that if the study reported a hazard ratio (HR) of SARS-CoV-2 RNA shedding [10–13] rather than viral clearance delay, then an HR of viral clearance delay was obtained by taking the reciprocal of the HR i.e.1/ HR and associated confidence interval (CI).

### Assessment of risk of bias

The Newcastle–Ottawa-Scale (NOS) [14] for observational studies and using the Revised Cochrane risk-of-bias tool for randomized trials (RoB 2) were used to assess the risk of bias by two independent groups of four authors (H.Y. and W.Z.; Y.Z. and LP.W.). Each domain of NOS was composed of 2 to 4 items of criteria, and each criterion was scored in the form of stars. A total score of 8 or 9 was assessed as low risk of bias, 6 or 7 as some concerns, and ≤ 5 as high risk. Each domain of RoB 2 was assessed as low risk, some concerns, or a high risk of bias. The study's overall risk of bias was determined by the highest risk of bias for any criteria. Disagreements were resolved by consensus, and if necessary, consultation with a third author (ZW.Z.).

### Data synthesis

Statistical analyses were performed using the meta package in R (version 4.0.1; The R Project for Statistical Computing). We mainly used risk ratios (RRs) and their associated 95% CI to assess outcomes, as well as a prediction interval (PI) for the effect of future studies based on the present [15]. Adjusted RRs and unadjusted RRs (including frequency counts in the 2 × 2 table for binary data) were separately pooled. We equivalently transformed HRs [16] and odds ratios (ORs) [17] into RRs and log-transform these effect sizes as well as their standard errors first before they were pooled. If provided, we also pooled mean difference (MD) for continuous data. We used random-effects models to pool data. The I^2^ test was used to examine heterogeneity and I^2^ ≥ 50% was considered as significant heterogeneity. A 2-tailed P value of less than 0.05 was statistically significant. Funnel plots and the Egger test were adopted to assess the publication bias of the main results.

### Subgroup analysis

We planned several subgroup analyses according to the following variables: (1) severity of illness (mild or moderate and severe or critical); (2) doses (equivalent methylprednisolone) of glucocorticoids (low dose [0.5–1 mg/kg/day or < 80 mg/day], medium dose [1–2 mg/kg/day or 80–200 mg/day], and high dose [> 2 mg/kg/day or > 200 mg/day]). The severity of illness was reported by the studies following Chinese interim guidelines for diagnosis and treatment for COVID-19 patients (version 7.0) [18, 19].

### Sensitivity analysis

We conducted sensitivity analyses on main results from adjusted RRs by (1) influence analyses [20] based on leave-one-out-method (2) excluding studies of case–control design, (3) excluding studies of retrospective cohort design, (4) excluding studies with the non-low risk of bias.

## Results

### Eligible studies and study characteristics

**Fig. 1:**
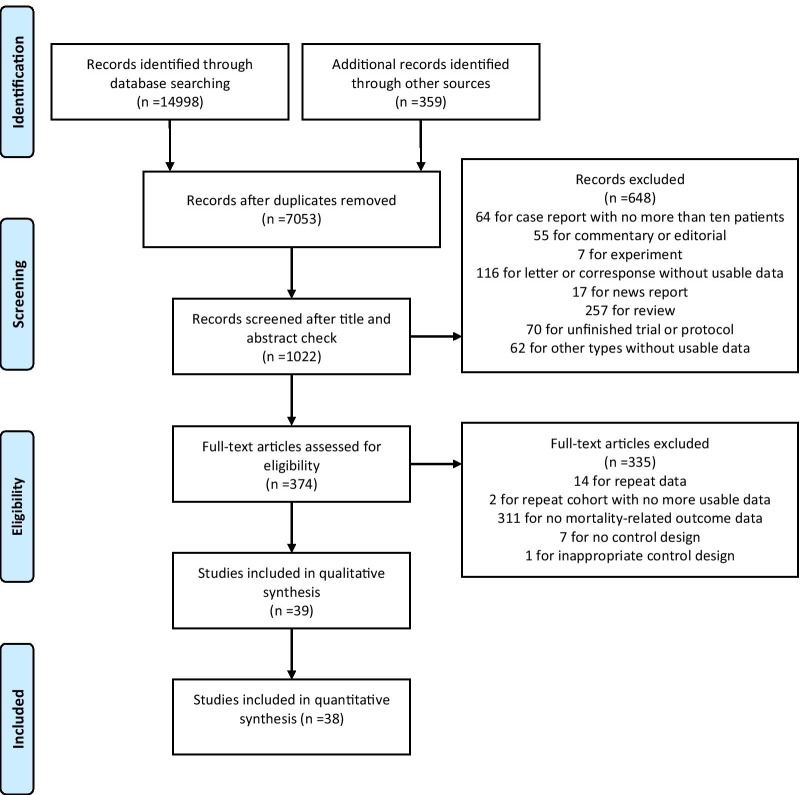
Preferred Reporting Items for Systematic Reviews and Meta-Analyses (PRISMA) for the Article Selection Process

Of the 15,357 records, 38 studies [6–13, 21–50] involving a total of 9572 patients were included in both qualitative and quantitative synthesis, and another study [51] was included only for qualitative synthesis (Fig. 1). Table 1 showed the characteristics of the included 39 studies. These studies, with a size from 31 to 966 and a median age from 39 to 66, comprised 1 RCT, 13 case–control studies, and 25 retrospective cohort studies. One of the studies came from Brazil, one from Spain, two from Italy, and the rest from China. Most studies used a low or medium dose of glucocorticoids, and a few studies [24, 40] reported the use of high-dose glucocorticoids. Methylprednisolone was the most common type, followed by dexamethasone, prednisone and prednisolone, and finally hydrocortisone. The median days for glucocorticoids treatment from illness onset ranged from 8 to 13 days and the median duration of treatment from 3 to 10.8 days. The studies reported different time frames of viral clearance delay, between 5- and 45-day, and the longest reported follow-up was 90 days.

**Table1:** Characteristics of Included Studies

Author, year	Country	Design	Size	Age (Median/median range, years)	Glucocorticoids dose (Equivalent of MP)	Glucocorticoids type	Treatment timing (From illness onset, median days)	Treatment duration (Median/range, days)	Time frame of viral clearance delay (Follow-up)
Cao and Zhu et al. [20]	China	CC	87	NA	NA	NA	NA	NA	14-, 10-day
Chang and Zhao et al. [21]	China	CC	67	14–59	NA	NA	NA	NA	16-day (16 days)
Chen and Li et al. [23]	China	RC	70	47–64	0.5–1 mg/kg/d (low dose); 1–2 mg/kg/d (medium-dose); 200–400 mg/d (high-dose)	NA	NA	NA	NA
Chen and Song et al. [22]	China	RC	371	63–65	Mean 49.5 mg/d	DM, MP, PN	Mean 14.1	Mean 9.1	NA
Chen and Zhu et al. [9]	China	RC	267	49	NA	NA	NA	NA	45-day
Cogliati-Dezza and Oliva et al. [5]	Italy	CC	179	62	NA	DM, MP	NA	NA	14-day
Ding and Feng et al. [24]	China	RC	82	49	NA	NA	NA	NA	NA
Fang and Mei et al. [25]	China	RC	55	39.9–60.6	Median 38 mg/d in general patients; 40 mg/d in severe patients	MP	NA	7 in general patients; 4.5 in severe patients	NA
Feng and Li et al. [6]	China	CC	564	47	NA	NA	NA	NA	NA (50 days)
Fu and Luo et al. [26]	China	RC	33	41–65	1 mg/kg/d	MP	NA	NA	NA (≥ 22 days)
Gong and Guan et al. [27]	China	RC	34	33.8–38.2	1–2 mg/kg/d	MP	NA	5–10	NA
Hu and Li et al. [10]	China	CC	206	Mean 53.7	40 mg/d (low-dose); 80 mg/d (high-dose)	MP	NA	NA	30-day
Hu and Yin et al. [28]	China	CC	183	49	Median 43.3 mg/d	NA	NA	4	20-day
Huang and Zhu et al. [11]	China	RC	309	45	40–160 mg/d	MP	NA	NA	NA (40 days)
Jeronimo and Farias et al. [29]	Brazil	RCT	283	Mean 55	1 mg/kg/day	MP	NA	5	5-, 7-day
Ji and Zhang et al. [30]	China	RC	490	61	1–2 mg/kg/day	DM, MP, PN	NA	3–5	14-, 28-day
Li and Cao et al. [31]	China	CC	66	47.5	NA	MP	NA	NA	11-day
Li and Li et al. [12]	China	RC	475	42	20 or 40 mg/d	MP, PS	2 (from admission)	NA	NA (50 days)
Li and Meng et al. [32]	China	RC	294	66	Median 40 mg/d	DM, MP, HC, PN	0 (from ICU admission)	9	NA (90 days)
Liang and Chen et al. [33]	China	RC	966	60	≤ 1–2 mg/kg/d	MP	NA	> 3	NA (≥ 50 days)
Liu and Li et al. [35]	China	RC	646	57	Median 80 mg/d	DM, MP, PN	13	NA	NA
Liu and Zhang et al. [34]	China	RC	774	64	Median 40 mg/d	MP, PS	1 (from admission)	6	30-day
Lu and Liu et al. [36]	China	RC	374	47–51	Median 220 mg (cumulative dose)	NA	≤ 5 (from admission)	4	NA (≥ 40 days)
Ma and Qi et al. [37]	China	RC	72	Mean 60	40 or 80 mg/d	MP	NA	3	NA
Ma and Zeng et al. [38]	China	RC	368	Mean 46.2	Median 56.6 mg/d	MP, PS	9	5	NA
Masia and Fernandez-Gonzalez et al. [39]	Spain	RC	77	63.5–71	250–500 mg/d	MP	NA	3	NA
Ni and Ding et al. [40]	China	RC	72	46–52	0.75–1.5 mg/kg/d	MP	NA	NA	NA
Qi and Yang et al. [41]	China	CC	147	42	NA	NA	NA	NA	17-day
Shi and Wu et al. [42]	China	CC	99	54	Median 60 mg/d	NA	8	NA	28-day
Shu and He et al. [43]	China	CC	83	43	NA	NA	NA	NA	16-day
Spagnuolo and Guffanti et al. [44]	Italy	RC	149	63.5	Median of 0.38 mg/kg/d	DM, MP, PN	1 (from admission)	9	14-, 14 to 28-, 28 to 40-, > 40-day
Wu and Hou et al. [45]	China	RC	382	Mean 60.7	NA	DM, MP, PN	NA	NA	NA
Xia and Xu et al. [46]	China	RC	49	NA	0.75–1.5 mg/kg/d	MP	≤ 3 (from admission)	NA	NA (≥ 10 days)
Xiong and Jin et al. [50]	China	RC	66	Mean 62	Median 400 mg (cumulative dose)	NA	9	NA	NA (≥ 30 days)
Xu and Chen et al. [47]	China	CC	113	52	0.5–1 mg/kg/d	MP	NA	NA	15-day
Yan and Liu et al. [7]	China	CC	120	52	NA	NA	NA	NA	23-day
Yuan and Xu et al. [8]	China	RC	132	43.7–52	Median 52.2 mg/d	MP	8.3	10.8	NA
Zha and Li et al. [48]	China	RC	31	39	40–80 mg/d	MP	NA	NA	NA
Zuo and Liu et al.. [49]	China	CC	181	Mean 44.3	NA	NA	NA	NA	21-day

CC, case control; RC, retrospective cohort; RCT, randomized controlled trial; DM, dexamethasone; MP, methylprednisolone; PN, prednisone; PS, prednisolone; HC, hydrocortisone; NA, not available

Additional files 2, 3, 4: Tables S2–S4 showed the risk bias of the included studies, and Additional file 5: Tables S5 listed adjusted factors in each included study. 12 studies were considered as low risk, 25 as some concerns, and 2 as high risk. The average score of total risk bias for case–control studies was 6.9 and the average score for retrospective cohort studies was 7.0. The only RCT was assessed as the trial with the risk bias of some concerns, due to its deviations from intended interventions.

### Risk of viral clearance delay

**Fig. 2:**
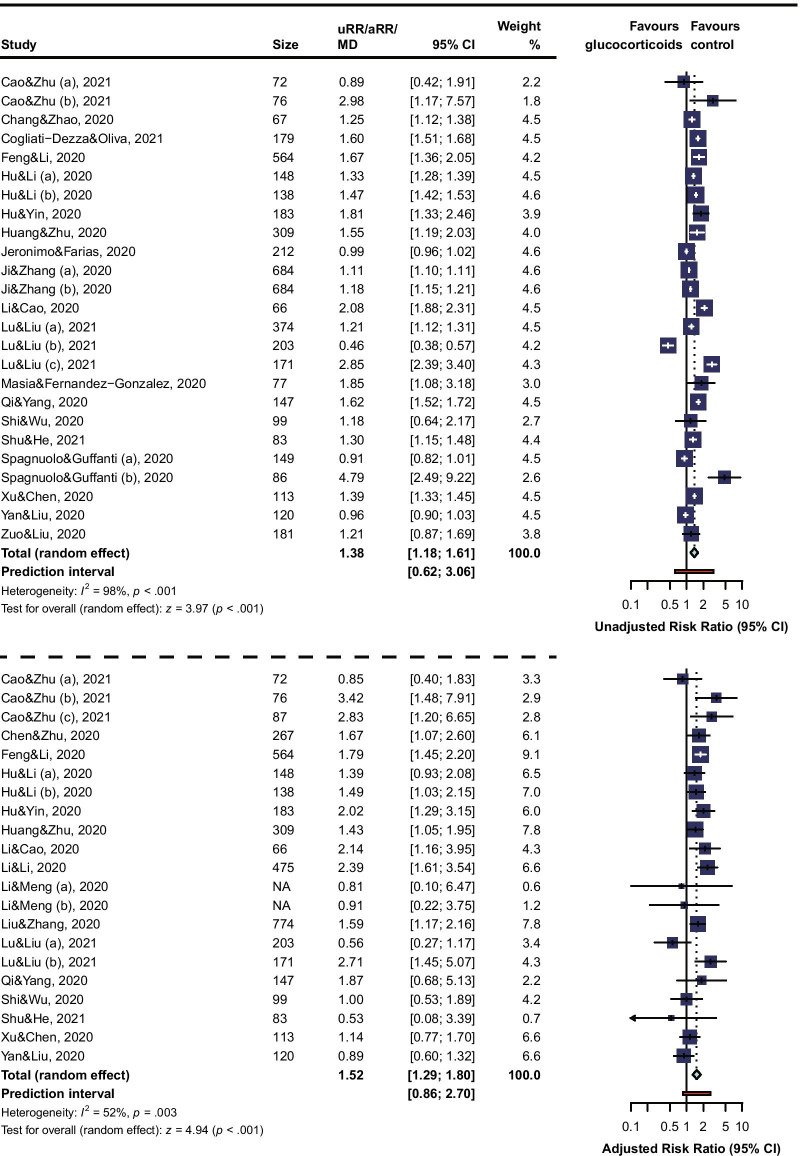

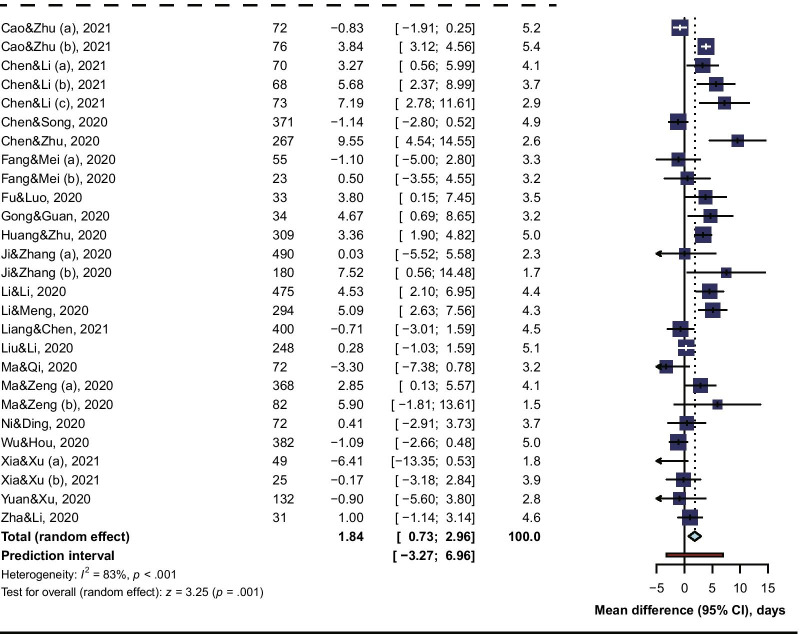
Forest Plot for Risk of Viral Clearance Delay. The “(a), (b), or (c)” indicates one study with subgroups data was included in the analysis

A total of 23 studies were involved to calculate RRs for risk of viral clearance delay in COVID-19 patients who received glucocorticoids treatment, of which the longest follow-up was 50 days. The overall unadjusted RR (1.38, 95% CI 1.18 to 1.61, I^2^ = 98%, PI 0.62 to 3.06) and adjusted RR (1.52, 95% CI 1.29 to 1.80, I^2^ = 52%, PI 0.86 to 2.70) (Fig. 2) revealed an association between glucocorticoids treatment and increased risk of viral clearance delay in COVID-19 patients. There were 20 studies reporting the time of viral shedding. The pooled MD of days for SARS-CoV-2 RNA shedding from illness onset (1.84, 95% CI 0.73 to 2.96, I^2^ = 83%, PI −3.27 to 6.96) (Fig. 2) also confirmed the delayed viral clearance in glucocorticoids treatment patients, compared to patients receiving non-glucocorticoids treatment.

Influence analyses found no study with an excessive influence on the overall results and the sensitivity analysis using the leave-one-out method confirmed the stability of the estimated adjusted RR (Additional file 6: Figure S1). The other sensitivity analyses also showed a similar result to that from the main analysis (Additional files 7, 8, 9: Figure S2–S4).

### Subgroup analysis

**Fig. 3:**
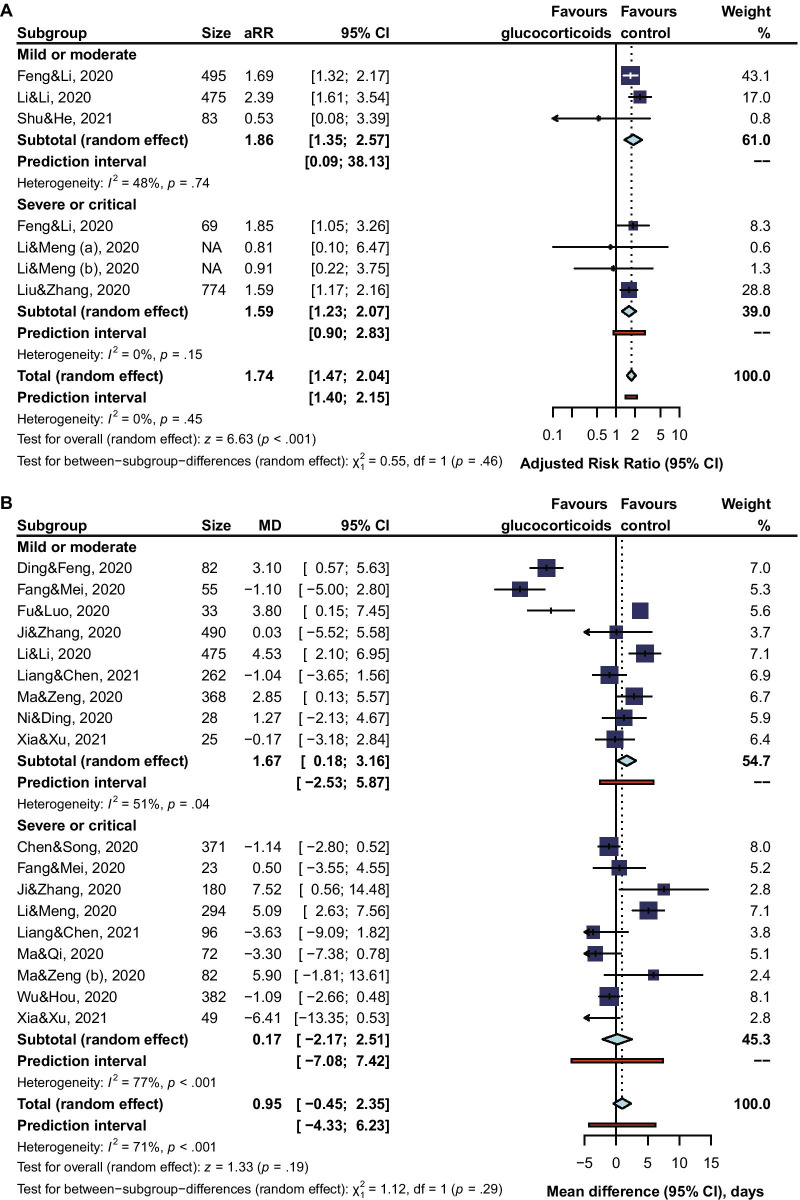
Subgroup Analysis by Severity of Illness

**Fig. 4:**
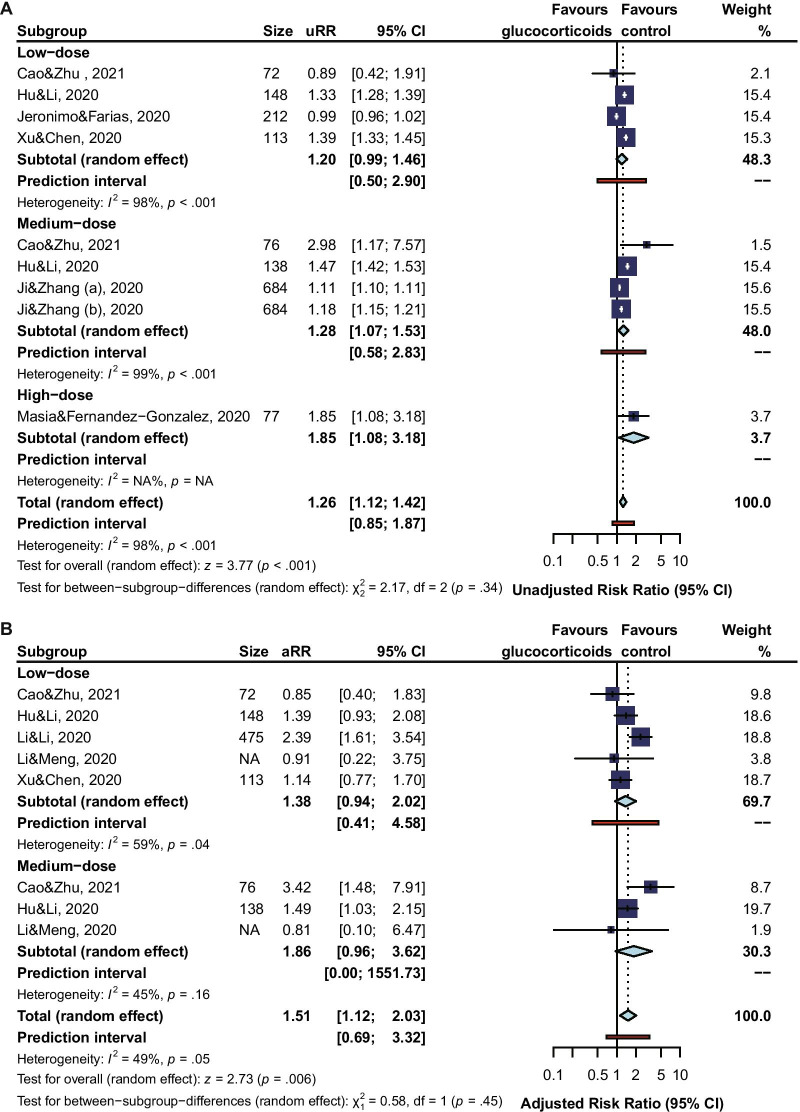

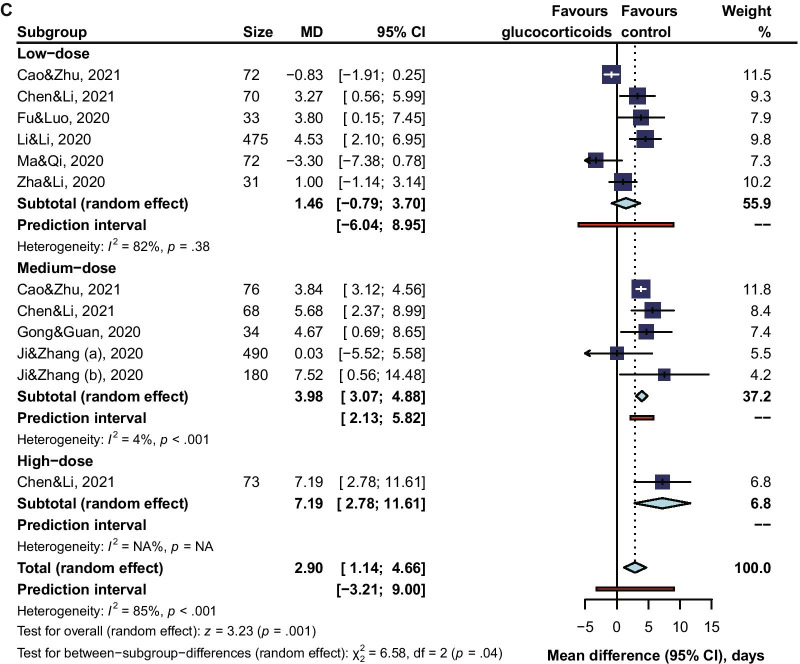
Subgroup Analysis by Doses of Glucocorticoids

Subgroup analysis revealed that the risk of viral clearance delay was significantly higher in glucocorticoids-treated COVID-19 patients of being mild or moderate (adjusted RR 1.86, 95% CI 1.35 to 2.57, I^2^ = 48%; MD 1.67, 95% CI 0.18 to 3.61, I^2^ = 24%), but not so high for patients of being severe or critical (adjusted RR 1.59, 95% CI 1.23 to 2.07, I^2^ = 0%; MD 0.95, 95% CI -2.71 to 2.51, I^2^ = 77%) (Fig. 3). When comparing the risk of viral clearance delay between a low dose, a medium dose, and a high dose of glucocorticoids, subgroup analysis showed that a high dose (unadjusted RR 1.85, 95% CI 1.08 to 3.18; MD 7.19, 95% CI 2.78 to 11.61) or a medium dose (unadjusted RR 1.28, 95% CI 1.07 to 1.53, I^2^ = 99%; adjusted RR 1.86, 95% CI 0.96 to 3.62, I^2^ = 45%; MD 3.98, 95% CI 3.07 to 4.88, I^2^ = 4%) of glucocorticoids increased the risk of viral clearance delay, but not for a low dose (unadjusted RR 1.20, 95% CI 0.99 to 1.46, I^2^ = 98%; adjusted RR 1.38, 95% CI 0.94 to 2.02, I^2^ = 59%; MD 1.46, 95% CI −0.79 to 3.70, I^2^ = 82%) (Fig. 4). In our qualitative synthesis, a few studies [33, 45, 47, 51] investigated the effect of use timing of glucocorticoids on the viral shedding, the results indicated late use rather than early use of glucocorticoids was significantly associated with a high risk of viral clearance delay.

### Publication bias

There was a significant asymmetry on the report of unadjusted RRs (Additional file 10: Figure S5), however, funnel plot analysis showed no asymmetry on the report of adjusted RRs and MDs (Additional files 11, 12: Figure S6–S7), and the Egger test detected no significant small-study effects.

## Discussion

In this meta-analysis of 38 studies (at moderate risk of bias involving 9572 patients), glucocorticoids treatment was significantly associated with an increased risk of viral clearance delay in COVID-19 patients. Evidence indicated a high or medium-dose but not a low dose of glucocorticoids, could substantially lead to viral clearance delay. Though only one RCT was included, however, adjusted data from observational studies and low heterogeneity of pooled data ensured the power of conclusions.

### Principal findings and comparison with other studies

As of writing this manuscript (early May 2021), no meta-analysis has quantitatively examined the use of glucocorticoids in patients with COVID-19 regarding viral clearance delay. Yousefifard et al.. conducted a meta-analysis on efficacy and safety of glucocorticoids on the management of COVID-19, SARS, and MERS, in which the search was conducted at the end of March 2020, and only 5 studies on COVID-19 were included [52]. Not enough data allowed that meta-analysis to quantitatively analyze glucocorticoids on the effect of viral clearance delay and the conclusion that corticosteroids administration would not be effective in shortening viral clearance time was only supported by qualitative analysis of evidence from a case report of nine COVID-19 patients, although it did not conflict with our conclusion in essence. Most trials of glucocorticoids suspended enrollment after the RECOVERY trial which was the globally largest one and drew an encouraging conclusion of reduction in mortality of COVID-19. However, as one kind of immunosuppressant, glucocorticoids’ detrimental effect-one of the most important side effects, i.e. viral clearance delay-had not been further investigated in these trials. Thus, information about its impact on the humoral immune response against the virus is in need. Most previous experience with patients infected by SARS, MERS, and H1N1 indicated that glucocorticoids delayed viral RNA clearance [53–55]. Nevertheless, one study on factors promoting the prolonged shedding of H1N1 indicated a significant association of viral clearance delay and delayed antiviral therapy, but not glucocorticoids treatment [56]. However, glucocorticoids treatment usually delayed antiviral therapy for two or more days after symptom onset and thus might have a more indirect role on the viral clearance delay [56]. Evidence is inconsistent on the viral clearance delay of glucocorticoid-treated COVID-19 patients. The most focus of the debate is the potential confounding role of doses and the severity of illness on the associations. Our meta-analysis pooled confounders-adjusted RRs and conducted subgroup analyses by doses and the severity of illness. The findings of our meta-analysis of the association of glucocorticoids administration with delayed viral RNA clearance were in line with the recently published results on COVID-19. We further discovered this association occurred in patients receiving a high or a medium dose, but not a low dose of glucocorticoids.

## Strengths and limitations

This systematic review and meta-analysis have several methodological strengths. We focused on the results of pooling adjusted RRs which could balance other potential confounders as possible. To exam the robustness of our main results, we conducted a series of presumable sensitivity analyses, in which extreme values were detected by influence analysis and then excluded by the leave-one-out method to avoid distortion of our pooled effect estimate. Moreover, we assessed potential high-risk subgroups by doses and the severity of illness, which was the main concern of glucocorticoids administration.

Our study has limitations. First, the results of this meta-analysis were main from observational studies and clinical heterogeneity was inevitable. Moreover, we failed to investigate the heterogeneity among studies that reported MDs due to limited data of confounders. Besides, the role of duration and types of glucocorticoids treatment on the viral clearance delay has not been further investigated due to insufficient accuracy of the information or lack of uniformity between studies. Moreover, most data came from China and whether the conclusion could be extended to other areas may be questionable. Finally, most of the reported data came from the early stage of the epidemic, and there were few reports on the data related to the mutated virus.

### Implications for practice

Through, people who have a lot of experience with glucocorticoids in the treatment of inflammatory, little information could be obtained regarding its role in the humoral immune response against the virus. Many years ago, one trial involving 29 normal adult males showed that short courses (3 or 5 days) of high dose (96 mg/d) methylprednisolone could decrease serum IgG concentration [57]. Theoretically, the reduction in antibody production might delay viral clearance and experience a high risk of reinfection [40]. However, there was one study that demonstrated that dexamethasone treatment did not affect the formation of pneumococcal antibodies during community-acquired pneumonia [58]. Viral pneumonia should be different from bacterial pneumonia. Previous studies on H1N1, SARS, MERS have shown glucocorticoids’ negative effects on viral clearance, however, the evidence is sporadic. We did the first meta-analysis to systematically investigate glucocorticoids’ role on the viral clearance delay of SARS-CoV-2. Our conclusion indicated glucocorticoids might delay viral clearance in patients taking a high or medium dose, but not in patients taking a low dose. We believe our findings would further bring light to the current clinical practice in glucocorticoids treatment of COVID-19. Of note, one aspect worth further studying involves those with the hypercoagulable condition of high D-dimer level, as a concerning risk factor of COVID-19 death. The therapeutic effect of different treatment methods may be affected by the D-dimer level. Di Castelnuovo et al.. found heparin use was associated with lower in-hospital mortality in COVID-19 patients with D-dimer > 2020 ng/mL [59], whereas our previous meta-analysis indicated D-dimer seemed not to affect the associations between glucocorticoids treatment and mortality of COVID-19 patients [60]. Mondi et al.. reported D-dimer > 1000 ng/mL at admission independently predicted prolonged SARS-CoV-2 RNA shedding [61]. However, whether the association between glucocorticoids treatment and viral clearance delay for COVID-19 patients is affected by D-dimer level or not is still unclear. Moreover, other details of glucocorticoids treatment also need to be further explored in the future, such as the timing, duration, species, etc., and even the immune status of the population and the variation of the virus.

## Conclusions

The findings suggest that glucocorticoids treatment delayed viral clearance in COVID-19 patients Moreover, it seems that patients taking a high dose or medium-dose rather than taking a low dose would experience a high risk of viral clearance delay.

## Supplementary Information

**Additional file 1: Table S1.** Search Strategy.

**Additional file 2: Table S2.** Risk of Bias of Case–control Studies by the Newcastle–Ottawa-Scale (NOS) Assessment.

**Additional file 3: Table S3.** Risk of Bias of Retrospective Cohort Studies by the Newcastle–Ottawa-Scale (NOS) Assessment.

**Additional file 4: Table S4.** Risk of Bias of the included RCT.

**Additional file 5: Table S5.** Adjusted Factors in Each Included Study.

**Additional file 6: Figure S1.** Sensitivity Analyses by Influence Analyses Based on Leave-one-out-method.

**Additional file 7: Figure S2.** Sensitivity Analyses by Excluding Studies of Case Control Design.

**Additional file 8: Figure S3.** Sensitivity Analyses by Excluding Studies of Retrospective Cohort Design.

**Additional file 9: Figure S4.** Sensitivity Analyses by Excluding Studies with Non-low Risk of Bias.

**Additional file 10: Figure S5.** Funnel Plot of Unadjusted Risk Ratios for Risk of Viral Clearance Delay.

**Additional file 11: Figure S6.** Funnel Plot of Adjusted Risk Ratios for Risk of Viral Clearance Delay.

**Additional file 12: Figure S7. **Funnel Plot of Mean Differences for Risk of Viral Clearance Delay.

## Data Availability

Additional data are available from the corresponding author on reasonable request at kangyan@scu.edu.cn.

## Abbreviations

COVID-19: Coronavirus disease 2019
SARS: Severe acute respiratory syndrome
RCT: Randomized controlled trial
MOOSE: A proposal for reporting meta-analysis of observational studies in epidemiology
PRISMA: Preferred reporting items for systematic reviews and meta-analyses
NOS: Newcastle–Ottawa-Scale
HRs: Hazard ratios
ORs: Odds ratios
RRs: Risk ratios
MD: Mean difference
CI: Confidence interval
PI: Prediction interval
