# Supplementary material for: Association between glucocorticoids treatment and viral clearance delay in patients with COVID-19: a systematic review and meta-analysis
Source: BMC Infect Dis. 2021 Oct 14;21:1063. doi: 10.1186/s12879-021-06548-z (PMC8514812; doi:10.1186/s12879-021-06548-z)
Supplement: Supplementary file 1 — Additional file 1: Table S1. Search Strategy. [file 12879_2021_6548_MOESM1_ESM.docx]

**Additional file 1:Table S1. Search Strategy**

| **1.        Medline (Ovid, 2019 to April 20, 2021) (n= 880 results)** |
| --- |
| 1 ("COVID19" or "COVID-19" or "coronavirus disease 2019" or "coronavirus disease-19" or "novel coronavirus" or "SARS-CoV-2" or "SARS2" or "2019-nCoV" or "2019 nCoV").mp. [mp=title, abstract, original title, name of substance word, subject heading word, floating sub-heading word, keyword heading word, organism supplementary concept word, protocol supplementary concept word, rare disease supplementary concept word, unique identifier, synonyms] |
| 2 ("glucocorticoid" or "corticosteroid" or "steroid" or "cortisone" or "hydrocortisone" or "prednisolone" or "methylprednisolone" or "prednisone" or "dexamethasone" or "triamcinolone").mp. [mp=title, abstract, original title, name of substance word, subject heading word, floating sub-heading word, keyword heading word, organism supplementary concept word, protocol supplementary concept word, rare disease supplementary concept word, unique identifier, synonyms] |
| 3 1 and 2 |
| **2.        Embase (Ovid, 2019 to April 20, 2021) (n= 4512 results)** |
| 1 ("COVID19" or "COVID-19" or "coronavirus disease 2019" or "coronavirus disease-19" or "novel coronavirus" or "SARS-CoV-2" or "SARS2" or "2019-nCoV" or "2019 nCoV").mp. [mp=title, abstract, original title, name of substance word, subject heading word, floating sub-heading word, keyword heading word, organism supplementary concept word, protocol supplementary concept word, rare disease supplementary concept word, unique identifier, synonyms] |
| 2 ("glucocorticoid" or "corticosteroid" or "steroid" or "cortisone" or "hydrocortisone" or "prednisolone" or "methylprednisolone" or "prednisone" or "dexamethasone" or "triamcinolone").mp. [mp=title, abstract, original title, name of substance word, subject heading word, floating sub-heading word, keyword heading word, organism supplementary concept word, protocol supplementary concept word, rare disease supplementary concept word, unique identifier, synonyms] |
| 3 1 and 2 |
| **3.        EBSCO (2019 to April 20, 2021) (n= 5895 results)** |
| S1 TX "COVID19" OR TX "COVID-19" OR TX "coronavirus disease 2019" OR TX "coronavirus disease-19" OR TX "novel coronavirus" OR TX "SARS-CoV-2" OR TX "SARS2" OR TX "2019-nCoV" OR TX "2019 nCoV" [Search modes: Boolean/Phrase] [Expanders: Apply related words & Also search within the full text of the articles & Apply equivalent subjects] |
| S2 TX "glucocorticoid" OR TX "corticosteroid" OR TX "steroid" OR TX "cortisone" OR TX "hydrocortisone" OR TX "prednisolone" OR TX "methylprednisolone" OR TX "prednisone" OR TX "dexamethasone" OR TX "triamcinolone" [Search modes: Boolean/Phrase] [Expanders: Apply related words & Also search within the full text of the articles & Apply equivalent subjects] |
| S3 S2 AND S1 |
| **4.        ScienceDirect (2019 to April 20, 2021) (n= 1345 results)** |
| 1. COVID AND ("glucocorticoid" OR "corticosteroid" OR "steroid" OR "cortisone" OR "hydrocortisone" OR "prednisolone" OR "methylprednisolone" OR "prednisone") [title, abstract or author-specified keywords] |
| 2. refine 1 by years: 2019-2021 |
| 3. COVID AND ("dexamethasone" OR "triamcinolone") [title, abstract or author-specified keywords] |
| 4. refine 3 by years: 2019-2021 |
| 5. coronavirus AND ("glucocorticoid" OR "corticosteroid" OR "steroid" OR "cortisone" OR "hydrocortisone" OR "prednisolone" OR "methylprednisolone" OR "prednisone") [title, abstract or author-specified keywords] |
| 6. refine 5 by years: 2019-2021 |
| 7. coronavirus AND ("dexamethasone" OR "triamcinolone") [title, abstract or author-specified keywords] |
| 8. refine 7 by years: 2019-2021 |
| 9. SARS-CoV AND ("glucocorticoid" OR "corticosteroid" OR "steroid" OR "cortisone" OR "hydrocortisone" OR "prednisolone" OR "methylprednisolone" OR "prednisone") [title, abstract or author-specified keywords] |
| 10. refine 9 by years: 2019-2021 |
| 11. SARS-CoV AND ("dexamethasone" OR "triamcinolone") [title, abstract or author-specified keywords] |
| 12. refine 11 by years: 2019-2021 |
| 13. SARS2 AND ("glucocorticoid" OR "corticosteroid" OR "steroid" OR "cortisone" OR "hydrocortisone" OR "prednisolone" OR "methylprednisolone" OR "prednisone") [title, abstract or author-specified keywords] |
| 14. refine 13 by years: 2019-2021 |
| 15. SARS2 AND ("dexamethasone" OR "triamcinolone") [title, abstract or author-specified keywords] |
| 16. refine 15 by years: 2019-2021 |
| 17. nCoV AND ("glucocorticoid" OR "corticosteroid" OR "steroid" OR "cortisone" OR "hydrocortisone" OR "prednisolone" OR "methylprednisolone" OR "prednisone") [title, abstract or author-specified keywords] |
| 18. refine 17 by years: 2019-2021 |
| 19. nCoV AND ("dexamethasone" OR "triamcinolone") [title, abstract or author-specified keywords] |
| 20. refine 19 by years: 2019-2021 |
| **5.        Web of Science (All database, 2019 to April 20, 2021) (n= 2083 results)** |
| #1 TS= ("COVID19" OR "COVID-19" OR "coronavirus disease 2019" OR "coronavirus disease-19" OR "novel coronavirus" OR "SARS-CoV-2" OR "SARS2" OR "2019-nCoV" OR "2019 nCoV") [Timespan= 2019-2021; Search language= Auto] |
| #2 TS= ("glucocorticoid" OR "corticosteroid" OR "steroid" OR "cortisone" OR "hydrocortisone" OR "prednisolone" OR "methylprednisolone" OR "prednisone" OR "dexamethasone" OR "triamcinolone") [Timespan= 2019-2021; Search language= Auto] |
| #3 #2 AND #1 [Databases= WOS, DIIDW, INSPEC, KJD, MEDLINE, RSCI, SCIELO; Timespan= 2019-2021; Search language= Auto] |
| **6.        Cochrane Library (2019 to April 20, 2021) (n= 250 results)** |
| #1 "COVID19" OR "COVID-19" OR "coronavirus disease 2019" OR "coronavirus disease-19" OR "novel coronavirus" OR "SARS-CoV-2" OR "SARS2" OR "2019-nCoV" OR "2019 nCoV" [in Title Abstract Keyword - (Word variations have been searched)] |
| #2 "glucocorticoid" OR "corticosteroid" OR "steroid" OR "cortisone" OR "hydrocortisone" OR "prednisolone" OR "methylprednisolone" OR "prednisone" OR "dexamethasone" OR "triamcinolone" [in Title Abstract Keyword - (Word variations have been searched)] |
| #3 #2 AND #1 |
| **7.        ClinicalTrials.gov (Commence to April 20, 2021) (n= 33 results)** |
| "COVID19" OR "COVID-19" OR "coronavirus disease 2019" OR "coronavirus disease-19" OR "novel coronavirus" OR "SARS-CoV-2" OR "SARS2" OR "2019-nCoV" OR "2019 nCoV" \| "glucocorticoid" OR "corticosteroid" OR "steroid" OR "cortisone" OR "hydrocortisone" OR "prednisolone" OR "methylprednisolone" OR "prednisone" OR "dexamethasone" OR "triamcinolone"[status=completed] |
