# Supplementary material for: Association between glucocorticoids treatment and viral clearance delay in patients with COVID-19: a systematic review and meta-analysis
Source: BMC Infect Dis. 2021 Oct 14;21:1063. doi: 10.1186/s12879-021-06548-z (PMC8514812; doi:10.1186/s12879-021-06548-z)
Supplement: Supplementary file 2 — Additional file 2: Table S2. Risk of Bias of Case–control Studies by the Newcastle–Ottawa-Scale (NOS) Assessment. [file 12879_2021_6548_MOESM2_ESM.docx]

**Additional file 2:Table S2. Risk of Bias of Case-control Studies by the Newcastle-Ottawa-Scale (NOS) Assessment**

| **Author** | **Is the case definition adequate** | **Representativeness of the cases** | **Selection of controls** | **Definition of controls** | **Comparability of cases and controls based on the design or analysis** | **Ascertainment of exposure** | **The same method of ascertainment for cases and controls** | **Non-response rate** | **Total score** | **Risk level** |
| --- | --- | --- | --- | --- | --- | --- | --- | --- | --- | --- |
| Cao&Zhu et al | 1 | 1 | 0 | 1 | 2 | 0 | 1 | 1 | 7 | Some concerns |
| Chang&Zhao et al | 1 | 1 | 0 | 1 | 0 | 0 | 1 | 1 | 5 | High |
| Cogliati-Dezza&Oliva et al | 1 | 1 | 0 | 1 | 2 | 1 | 1 | 1 | 8 | Low |
| Feng&Li et al | 1 | 1 | 0 | 1 | 2 | 0 | 1 | 1 | 7 | Some concerns |
| Hu&Li et al | 1 | 1 | 0 | 1 | 1 | 1 | 1 | 1 | 7 | Some concerns |
| Hu&Yin et al | 1 | 1 | 0 | 1 | 2 | 1 | 1 | 1 | 8 | Low |
| Li&Cao et al | 1 | 1 | 0 | 1 | 2 | 1 | 1 | 1 | 8 | Low |
| Qi&Yang et al | 1 | 1 | 0 | 1 | 2 | 0 | 1 | 1 | 7 | Some concerns |
| Shi&Wu et al | 1 | 1 | 0 | 1 | 1 | 1 | 1 | 1 | 7 | Some concerns |
| Shu&He et al | 1 | 1 | 0 | 1 | 1 | 0 | 1 | 1 | 6 | Some concerns |
| Xu&Chen et al | 1 | 1 | 0 | 1 | 2 | 1 | 1 | 1 | 8 | Low |
| Yan&Liu et al | 1 | 1 | 0 | 1 | 2 | 0 | 1 | 1 | 7 | Some concerns |
| Zuo&Liu et al | 1 | 1 | 0 | 1 | 0 | 0 | 1 | 1 | 5 | High |
