# Supplementary material for: Association between glucocorticoids treatment and viral clearance delay in patients with COVID-19: a systematic review and meta-analysis
Source: BMC Infect Dis. 2021 Oct 14;21:1063. doi: 10.1186/s12879-021-06548-z (PMC8514812; doi:10.1186/s12879-021-06548-z)
Supplement: Supplementary file 3 — Additional file 3: Table S3. Risk of Bias of Retrospective Cohort Studies by the Newcastle–Ottawa-Scale (NOS) Assessment. [file 12879_2021_6548_MOESM3_ESM.docx]

**Additional file 3:Table S3. Risk of Bias of Retrospective Cohort Studies by the Newcastle-Ottawa-Scale (NOS) Assessment**

| **Author** | **Representative-ness of the exposed cohort** | **Selection of the non-exposed cohort** | **Ascertainment of exposure** | **Demonstration that outcome of interest was not present at the start of the study** | **Comparability of cohorts based on the design or analysis** | **Assessment of outcome** | **Was follow-up long enough for outcomes to occur** | **Adequacy of follow up of cohorts** | **Total score** | **Risk**  **level** |
| --- | --- | --- | --- | --- | --- | --- | --- | --- | --- | --- |
| Chen&Li et al | 1 | 1 | 1 | 1 | 0 | 1 | 0 | 1 | 6 | Some concerns |
| Chen&Song et al | 1 | 1 | 1 | 1 | 0 | 1 | 0 | 1 | 6 | Some concerns |
| Chen&Zhu et al | 1 | 1 | 0 | 1 | 2 | 1 | 1 | 1 | 8 | Low |
| Ding&Feng et al | 1 | 1 | 0 | 1 | 1 | 1 | 0 | 1 | 6 | Some concerns |
| Fang&Mei et al | 1 | 1 | 1 | 1 | 0 | 1 | 0 | 1 | 6 | Some concerns |
| Fu&Luo et al | 1 | 1 | 1 | 1 | 0 | 1 | 1 | 1 | 7 | Some concerns |
| Gong&Guan et al | 1 | 1 | 1 | 1 | 0 | 1 | 0 | 1 | 6 | Some concerns |
| Huang&Zhu et al | 1 | 1 | 1 | 1 | 2 | 0 | 1 | 1 | 8 | Low |
| Ji&Zhang et al | 1 | 1 | 1 | 1 | 0 | 1 | 1 | 1 | 7 | Some concerns |
| Li&Li et al | 1 | 1 | 1 | 1 | 2 | 0 | 1 | 1 | 8 | Low |
| Li&Meng et al | 1 | 1 | 1 | 1 | 2 | 1 | 1 | 1 | 9 | Low |
| Liang&Chen et al | 1 | 1 | 1 | 1 | 2 | 1 | 1 | 1 | 9 | Low |
| Liu&Li et al | 1 | 1 | 1 | 1 | 2 | 0 | 0 | 1 | 7 | Some concerns |
| Liu&Zhang et al | 1 | 1 | 1 | 1 | 1 | 1 | 1 | 1 | 8 | Low |
| Lu&Liu et al | 1 | 1 | 1 | 1 | 2 | 1 | 1 | 1 | 9 | Low |
| Ma&Qi et al | 1 | 1 | 1 | 1 | 0 | 1 | 0 | 1 | 6 | Some concerns |
| Ma&Zeng et al | 1 | 1 | 1 | 1 | 1 | 1 | 0 | 1 | 7 | Some concerns |
| Masia&Fernandez-Gonzalez et al | 1 | 1 | 1 | 1 | 0 | 0 | 1 | 1 | 6 | Some concerns |
| Ni&Ding et al | 1 | 1 | 1 | 1 | 0 | 1 | 0 | 1 | 6 | Some concerns |
| Spagnuolo&Guffanti et al | 1 | 1 | 1 | 1 | 2 | 1 | 1 | 1 | 9 | Low |
| Wu&Hou et al | 1 | 1 | 1 | 1 | 0 | 1 | 0 | 1 | 6 | Some concerns |
| Xia&Xu et al | 1 | 1 | 1 | 1 | 0 | 1 | 1 | 1 | 7 | Some concerns |
| Xiong&Jin et al | 1 | 1 | 1 | 0 | 0 | 1 | 1 | 1 | 6 | Some concerns |
| Yuan&Xu et al | 1 | 1 | 1 | 1 | 0 | 1 | 0 | 1 | 6 | Some concerns |
| Zha&Li et al | 1 | 1 | 1 | 1 | 0 | 1 | 0 | 1 | 6 | Some concerns |
