# Supplementary material for: Association between glucocorticoids treatment and viral clearance delay in patients with COVID-19: a systematic review and meta-analysis
Source: BMC Infect Dis. 2021 Oct 14;21:1063. doi: 10.1186/s12879-021-06548-z (PMC8514812; doi:10.1186/s12879-021-06548-z)
Supplement: Supplementary file 4 — Additional file 4: Table S4. Risk of Bias of the included RCT. [file 12879_2021_6548_MOESM4_ESM.docx]

**Additional file 4:Table S4. Risk of Bias of the included RCT**

| **Unique ID** | Jeronimo&Farias et. al^1^ | **Study ID** | NCT04343729 | **Assessor** | JB. L |
| --- | --- | --- | --- | --- | --- |
| **Ref or Label** | http://dx.doi.org/10.1093/cid/ciaa1177 | **Aim** | Assignment to intervention (the intention-to-treat effect) |  |  |
| **Experimental** | Methylprednisolone | **Comparator** | Placebo | **Source** | Journal article(s) with results of the trial; Trial protocol |
| **Outcome** | RR | **Results** | 0.99 | **Weight** | 1 |
| **Domain** | **Signaling question** | | | **Response** | **Comments** |
| **Bias arising from the randomization process** | 1.1 Was the allocation sequence random? | | | Y | An independent statistician prepared an electronically generated randomization list with 14 blocks of 30 participants per block, generated via R software version 3.6.1. The list was accessible only to nonblinded pharmacists in the study. Participants were randomized by the study pharmacist to their designated treatment regimen at the time of inclusion and were subsequently identified throughout the study only by their allocated study number. Nonblinded pharmacists prepared the dilutions in the wards and distributed syringes to the nursing staff labeled as MP/placebo. |
|  | 1.2 Was the allocation sequence concealed until participants were enrolled and assigned to interventions? | | | Y |  |
|  | 1.3 Did baseline differences between intervention groups suggest a problem with the randomization process? | | | N | There were no major differences in baseline characteristics between intervention and placebo groups (Table 1). |
|  | **Risk of bias judgment** | | | **Low** |  |
| **Bias due to deviations from intended interventions** | 2.1. Were participants aware of their assigned intervention during the trial? | | | N | An independent statistician prepared an electronically generated randomization list with 14 blocks of 30 participants per block, generated via R software version 3.6.1. The list was accessible only to nonblinded pharmacists in the study. Participants were randomized by the study pharmacist to their designated treatment regimen at the time of inclusion and were subsequently identified throughout the study only by their allocated study number. Nonblinded pharmacists prepared the dilutions in the wards and distributed syringes to the nursing staff labeled as MP/placebo. |
|  | 2.2. Were carers and people delivering the interventions aware of participants' assigned intervention during the trial? | | | Y |  |
|  | 2.3. If Y/PY/NI to 2.1 or 2.2: Were there deviations from the intended intervention that arose because of the experimental context? | | | N |  |
|  | 2.4 If Y/PY to 2.3: Were these deviations likely to have affected the outcome? | | | NA |  |
|  | 2.5. If Y/PY/NI to 2.4: Were these deviations from intended intervention balanced between groups? | | | NA |  |
|  | 2.6 Was an appropriate analysis used to estimate the effect of assignment to intervention? | | | Y | A modified intention to treat (MITT) analysis was conducted (all patients who have used at least one dose of the investigational drug, even with protocol deviations were included). |
|  | 2.7 If N/PN/NI to 2.6: Was there potential for a substantial impact (on the result) of the failure to analyses participants in the group to which they were randomized? | | | NA |  |
|  | **Risk of bias judgment** | | | **Some concerns** |  |
| **Bias due to missing outcome data** | 3.1 Were data for this outcome available for all, or nearly all, participants randomized? | | | Y |  |
|  | 3.2 If N/PN/NI to 3.1: Is there evidence that the result was not biased by missing outcome data? | | | NA |  |
|  | 3.3 If N/PN to 3.2: Could missingness in the outcome depend on its true value? | | | NA |  |
|  | 3.4 If Y/PY/NI to 3.3: Is it likely that missingness in the outcome depended on its true value? | | | NA |  |
|  | **Risk of bias judgment** | | | **Low** |  |
| **Bias in measurement of the outcome** | 4.1 Was the method of measuring the outcome inappropriate? | | | N | Two nasopharyngeal or one oropharyngeal swab (per institutional protocol) were used to extract viral RNA with the QIAAMP Viral RNA mini kit according to the manufacturer’s recommendations. Subsequently, all swab specimens were tested for SARS-COV-2 using the one-step multiple RT-QPCR kits produced by Instituto de Biological Molecular do Parana (IBMP, Curitiba, Brazil), following the manufacturer's recommendations and targeting the virus nucleocapsid (N) (HEX) and ORF-1AB (FA) genes and an endogenous human gene as the internal control (ROX). For all assays, specimens were considered positive if both viral targets, N1 and N2, showed cycle thresholds (CT) lower than 40.0. Swab specimens were collected on days 1 and 7. |
|  | 4.2 Could measurement or ascertainment of the outcome have differed between intervention groups? | | | N |  |
|  | 4.3 Were outcome assessors aware of the intervention received by study participants? | | | PN |  |
|  | 4.4 If Y/PY/NI to 4.3: Could assessment of the outcome have been influenced by knowledge of intervention received? | | | NA |  |
|  | 4.5 If Y/PY/NI to 4.4: Is it likely that assessment of the outcome was influenced by knowledge of intervention received? | | | NA |  |
|  | **Risk of bias judgment** | | | **Low** |  |
| **Bias in selection of the reported result** | 5.1 Were the data that produced this result analyzed following a pre-specified analysis plan that was finalized before unblinded outcome data were available for analysis? | | | Y | This study was conducted following the principles of the Declaration of Helsinki and the Good Clinical Practice guidelines of the International Conference on Harmonization. The protocol was approved by the Brazilian Committee of Ethics in Human Research. Random online clinical monitoring and quality control were performed. An independent Data and Safety Monitoring Board (DSMB), with intensive care clinicians and experts in infectious diseases, was set up to review preliminary and final analyses. The trial was reported according to the Consolidated Standards of Reporting Trials (Consort) guideline. The detailed protocol is available as Supplement 1. |
|  | 5.2 ... multiple eligible outcome measurements (e.g. scales, definitions, time points) within the outcome domain? | | | N |  |
|  | 5.3 ... multiple eligible analyses of the data? | | | N |  |
|  | **Risk of bias judgment** | | | **Low** |  |
| **Overall bias** | **Risk of bias judgment** | | | **Some concerns** |  |

1. Jeronimo CMP, Farias MEL, Val FFA, et al. Methylprednisolone as adjunctive therapy for patients hospitalized with COVID-19 (Metcovid): A randomized, double-blind, phase IIb, placebo-controlled trial. *Clinical Infectious Diseases* 2020.
