# Supplementary material for: Association between glucocorticoids treatment and viral clearance delay in patients with COVID-19: a systematic review and meta-analysis
Source: BMC Infect Dis. 2021 Oct 14;21:1063. doi: 10.1186/s12879-021-06548-z (PMC8514812; doi:10.1186/s12879-021-06548-z)
Supplement: Supplementary file 5 — Additional file 5: Table S5. Adjusted Factors in Each Included Study. [file 12879_2021_6548_MOESM5_ESM.docx]

**Additional file 5:Table S5. Adjusted Factors in Each Included Observational Study**

| **Author** | **Adjusted factors** |
| --- | --- |
| Chen&Zhu et al | Variables with a p-value of less than 0.05 in the univariate test were included in the multivariable Cox regression model (Age, Time from illness onset to hospital admission (days), Severity of disease, Diarrhea, Albumin (g/l), Lactic acid (mmol/l), ICU care, Oxygen therapy, Antibiotics, Lopinavir/ritonavir) |
| Feng&Li et al | Age, Sex et al |
| Huang&Zhu et al | Variables having P values < 0.1 in the univariate analysis were further used for a multivariate Cox regression analysis |
| Liu&Zhang et al | Competing events (death) |
| Ma&Zeng et al | Age (years) Sex, Male, and Any comorbidity |
| Hu&Yin et al | Variables with a P value of less than 0.05 in the univariate analysis and met the proportional hazard assumption (Cough, Sputum, Time from illness onset to hospital admission, Duration of viral shedding, Illness severity status, Radiographic extent, Lymphocyte count, N/L ratio, Albumin (g/L), Hs-CRP (mg/L), Antibiotics, Arbidol, Oseltamivir, Immunoglobulin) |
| Chen&Song et al | Not available |
| Hu&Li et al | Not available |
| Li&Cao et al | Age, Disease severity, T max (temperature), Time from onset to hospitalization |
| Yuan&Xu et al | Not available |
| Gong&Guan et al | Not available |
| Fu&Luo et al | Not available |
| Li&Li et al | Adjustment for differences in baseline characteristics of patients between the corticosteroids group and non-corticosteroids group using PSM |
| Xu&Chen et al | Age, Male sex, Hypertension Corticosteroid, Time from illness onset to hospitalization, Days Patients with severe disease at admission, Critical illness during hospitalization Invasive mechanical ventilation |
| Ding&Feng et al | Adjusted by Age, Sex, Invasive mechanical ventilation, and Progression to severe or critical |
| Chen&Li et al | Not available |
| Shi&Wu et al | Age and sex |
| Ni&Ding et al | Not available |
| Xia&Xu et al | Not available |
| Yan&Liu et al | Age, Sex, Current smoking, Hypertension, Cardiac disease, Diabetes, Corticosteroid, Lack of lopinavir/ritonavir |
| Qi&Yang et al | Factors that were statistically significant in the univariate analysis were further entered into the multivariate logistic regression analysis (Expectoration, Hemoptysis Highest temperature, Time from symptom onset to admission PaO2, Lymphocyte count <0.8*10⁹/L, Serum ferritin >300 mg/L, Glucocorticoids, Immunoglobulin, Hospital length of stay) |
| Ji&Zhang et al | Not available |
| Masia&Fernandez-Gonzalez et al | Not available |
| Zuo&Liu et al | Not available |
| Fang&Mei et al | Not available |
| Liu&Li et al | Only variables with a P-value <0.05 in univariable analysis or a presumptive association with the event were included to avoid overfitting. Moreover, considering patients were treated with corticosteroids at different times after admission, The treatment was taken as a time-varying covariate |
| Wu&Hou et al | Not available |
| Jeronimo&Farias  et al | Not available |
| Chang&Zhao et al | Not available |
| Zha&Li et al | Not available |
| Ma&Qi et al | Not available |
| Lu&Liu et al | Model adjusted for Gender, Hypertension, Diabetes, Oxygen saturation at admission, Symptom count at admission, Lopinavir/ritonavir, Oseltamivir, and Umifenovir use |
| Cogliati-Dezza&Oliva et al | Age, Sex, Tocilizumab, Steroids, ICU transfer, Albumin, NLR |
| Xiong&Jin et al | Variables with p < 0.1 were included as independent risk factors in the generalized linear model |
| Cao&Zhu et al | A time-dependent Cox proportional hazards model that adjusted for baseline covariates |
| Shu&He et al | Adjusted for Corticosteroids, Pneumonia, Delay in admission, and Comorbidities |
| Spagnuolo&Guffanti et al | Not available |
| Li&Meng et al | Adjustment for baseline differences and time-varying confounders (indication bias and immortal time bias). Time-varying variables were determined as PaO2/FiO2, Ventilation status, and SOFA scores on the day of corticosteroid therapy initiation. |
| Liang&Chen et al | A propensity score (PS) was estimated by logistic regression to determine the probability of corticosteroids treatment of each patient and the caliper was set to 0.02 |
